# Exploring well-being services from the perspective of people with SCI: A scoping review of qualitative research

**DOI:** 10.1080/17482631.2021.1986922

**Published:** 2021-10-25

**Authors:** Bronwyn Simpson, Michelle Villeneuve, Shane Clifton

**Affiliations:** a Occupational Therapy, Faculty of Medicine and Health, The University of Sydney, Sydney, Australia; b Centre for Disability Research, The University of Sydney, Sydney, Australia; c Royal Commission into Violence, Abuse, Neglect and Exploitation of People with Disability, Australia; Centre for Disability Research, The University of Sydney, Sydney, Australia

**Keywords:** Well-being, quality of life, spinal cord injury, rehabilitation, intervention, qualitative scoping review

## Abstract

**Objective:**

Well-being after spinal cord injury is affected by a range of factors, many of which are within the influence of rehabilitation services. Although improving well-being is a key aim of rehabilitation, the literature does not provide a clear path to service providers who seek to improve well-being. This study aimed to inform service design by identifying the experience and perspective of people with SCI about interventions targeting their well-being.

**Method:**

The scoping review of qualitative literature used thematic analysis to identify and categorize themes related to service activities, valued aspects, limitations and perceived outcomes.

**Results:**

Thirty-eight studies were selected, related to a range of service types. Most studies did not adopt a well-being conceptual framework to design and evaluate the services. People with SCI particularly valued being treated with dignity, positive expectations, increased autonomy and peer support. Improvements to well-being were reported, including many years post-SCI. However, people with SCI reported limited opportunities to engage in such services.

**Conclusions:**

Rehabilitation services can improve well-being across the lifetime of people with SCI, but gaps in service provision are reported. The review identified valued aspects of services that may inform service design, including staff approach and positive expectations, having own skills and worth valued, peer support and interaction, autonomy in valued occupations, and long-term opportunities for gains.

## Introduction

Maximizing well-being is a key focus of disability and rehabilitation services (Bertisch et al., 2015; Hammell, 2006; Pain et al., 1998; Whiteneck & Hall, 1992), at least in theory. According to the World Health Organization, the purpose of rehabilitation is to enable people “of all ages to maintain or return to their daily life activities, fulfil meaningful life roles and maximize their well-being” (World Health Organization, 2019) para. 1. Spinal cord injury (SCI) can negatively impact well-being (especially in the short to medium term), and is commonly thought to make life no longer worth living (an ableist assumption shared with many other disabilities) (Albrecht & Devlieger, 1999; Brickman et al., 1978; Peña-Guzmán & Reynolds, 2019). However, the impact of SCI on well-being is not straightforward. SCI can reduce well-being in a number of ways (Boakye et al., 2012; Dijkers, 1997; Murray et al., 2007), but many people with the injury experience post-traumatic growth (Bonanno et al., 2012; Byra, 2016; Griffiths & Kennedy, 2012; Kennedy et al., 2013; Pollard & Kennedy, 2007) and report that their lives are meaningful and satisfying (Albrecht & Devlieger, 1999; Bach & Tilton, 1994; Bonanno et al., 2012; Migliorini & Tonge, 2009).

The complex phenomenon of well-being appears to be influenced by a range of factors after SCI. Qualitative research has identified determinants that people with SCI perceive affecting their well-being (Bergmark et al., 2008; Clifton et al., 2018; Duggan et al., 2016; Geard et al., 2018; Hammell, 2007; Simpson et al., 2020). They report that their well-being is enhanced by the ability to engage in occupations, enjoy meaningful relationships, employ their strengths and values and take control of their daily life. These elements facilitate self-worth and self-continuity. They also report that body problems, a sense of loss and environmental barriers negatively impact their well-being. Making changes to these determinants of well-being is within the scope of rehabilitation and disability services (Simpson et al., 2020), and improving well-being should be a focus of service design and evaluation for people with SCI.

Well-being is defined poorly (if at all) in disability and rehabilitation research (Dijkers, 2005; Hill et al., 2010; Post, 2014; Simpson et al., 2020). A range of terms are used somewhat interchangeably with well-being in this body of literature (Svensson & Hallberg, 2011), including quality of life (Hill et al., 2010; Post, 2014; Tate et al., 2002), subjective well-being (Fuhrer, 2000), flourishing (Clifton et al., 2018), wellness (Carroll et al., 2020; Hall et al., 2021) and life satisfaction (Dunnum, 1990). Finding a clear and useful definition for these terms is also difficult. Broad definitions, such as ‘a life worth living’ (Csikszentmihalyi & Csikszentmihalyi, 2006; Janning, 2013; Migliorini et al., 2013; Seligman, 2011), capture the multidimensional nature of well-being, but these definitions may be too vague to be a guide for service design and evaluation. Narrower conceptions such as ‘health-related quality of life’ may miss aspects of well-being that are unrelated to, or unaffected by, a health condition, particularly for people with long-term disability like SCI. The complexity of conceptualizing well-being is exacerbated by the debate about whether the good life consists of subjective well-being (positive emotions, life satisfaction) or psychological well-being (meaning, character,growth) (Henderson & Knight, 2012; Kashdan et al., 2008; Keyes et al., 2002; Ryan & Deci, 2001). Rehabilitation service design and evaluation may be best guided by frameworks that list a range of well-being elements. Post (Post, 2014) has evaluated broad conceptual frameworks that may be used in disability service design and research. The elements of well-being proposed by positive psychology (positive emotions, engagement, relationships, meaning, accomplishment) (Seligman, 2011) are also a useful contribution to our understanding of well-being. However, these frameworks have not yet been widely adopted in rehabilitation and disability research (Shogren, 2013).

The lack of clarity about well-being is reflected in the tools used to measure this phenomenon for people with disability. There has been a positive trend towards including an outcome measure of well-being or quality of life in rehabilitation intervention studies, usually as a secondary measure. But questions have been raised about the suitability of commonly used measures of well-being, which are often designed by researchers without disability, for the general population (Amundsen, 2005; Dale, 1995; Dijkers, 1999; Hammell, 2004; Mackenzie & Scully, 2007; Slevin et al., 1988) rather than for people with SCI. Such measures have been criticized for overlooking well-being elements that may be important to people with long-term impairments and for over-emphasizing activities such as walking that may not be important for the well-being of people with SCI (Leplege & Hunt, 1997; Michel et al., 2016; Tate et al., 2002; Whitehurst et al., 2014). Thus, quantitative studies that adopt such well-being measures may not adequately reflect the priorities and experience of people with SCI. Furthermore, quantitative intervention studies do not provide an in-depth understanding of how or why well-being was enhanced.

Because well-being is a broad phenomenon influenced by a range of factors, it is possible that most services for people with SCI can influence well-being in some way. However, a more explicit focus on well-being may maximize the impact of these services, particularly when accompanied by efforts to measure their effectiveness (Hammell, 2006, 2017; Pizzi & Richards, 2017; Simpson et al., 2020). Intentionally designing services to address well-being requires an in-depth understanding of this phenomenon. However, there is a lack of clarity about how to define, address and measure the well-being of people with SCI. Consequently, SCI service providers who seek further understanding in the literature face a confusing maze. An in-depth understanding about well-being of people with SCI should be informed by the voices of people living with this condition. Several qualitative studies have sought the perspective of people with SCI about rehabilitation services related to their well-being. Understanding how people with SCI experience these services, including valued aspects, limitations and perceived outcomes, may help inform service design and evaluation. This paper sought to examine the perspective of people with SCI on services that addressed their well-being and to map and synthesize the qualitative literature on the topic.

## Aims

The larger aim of this paper is to give service providers insight into how to improve the delivery of their services by identifying the experience and perspective of people with SCI about interventions targeting their well-being. The specific aims were to i) examine the extent and nature of qualitative research related to well-being programs for people with SCI; ii) describe how well-being is conceptualized in these studies, and whether/how intentional design for well-being was used; iii) describe specific activities, timing and context of rehabilitation services related to well-being; and iv) explore how people with SCI perceive and experience these services. In collating this information from rich qualitative studies, the larger aim of this paper is to give service providers insight into how to improve the delivery of their services and maximize their participants’ well-being.

## Method

We used a scoping review methodology, which is well-suited to exploring the scope of research activity (Arksey & O’Malley, 2005; Rumrill et al., 2010), particularly for an emerging body of research about a poorly defined construct, which this appeared to be. We used the five stages proposed by Arksey & O’Malley (2005): 1) identifying the research question; 2) locating relevant studies; 3) selecting appropriate studies; 4) charting the data and 5) collating, summarizing and reporting the results.

## Stage 1: Identifying the research question

The overall question guiding this scoping review was as follows: “What is known from the existing qualitative literature about well-being services for people with SCI?” Our specific focus on SCI was guided by the assumption that well-being issues would be unique and specific to this population, due to the (usually) sudden onset of significant impairment. We acknowledged the complexities in defining well-being and included studies that referred to well-being (or a related concept such as quality of life) regardless of definition. Because well-being is multidimensional, we were also interested in studies that addressed a specific element of well-being or a specific outcome that is known to promote well-being. ‘Rehabilitation service’ is a similarly hard concept to define. We were guided by the World Health Organization’s definition of rehabilitation as a “*set of interventions needed when a person is experiencing or is likely to experience limitations in everyday functioning*” aiming to enable “*individuals of all ages to maintain or return to their daily life activities, fulfil meaningful life roles and maximize their well-being*” (World Health Organization, 2019). Rehabilitation services were defined as any activities, services or programs that appeared to promote these rehabilitation aims.

## Stage 2: Locating relevant studies

We conducted a database search of Medline, ADMED, Cochrane Database of Systematic Reviews, PsychARTICLES, PsychINFO, Embase and CINAHL. Search terms related to *spinal cord injury* were spinal cord injuries, spinal cord injur*, paraplegi*, tetraplegi*, and quadriplegi*. Search terms related to *well-being* were quality of life, personal satisfaction, wellbeing, well being, well-being, happiness, good life, wellness and flourish*. ‘Qualitative’ and ‘interview’ were also used as search terms to narrow the search to qualitative or mixed-methods studies, as our research question sought an in-depth perspective of people with SCI. Reference lists of included studies were hand searched to identify studies missed by the database search. Identifying these search terms was an iterative process, and we redefined search terms as early searches identified additional terms that were relevant.

## Stage 3: Selecting appropriate studies

Studies needed to include people with SCI of any cause or level, and of any age, but could also include people with conditions other than SCI. Included studies needed to relate to well-being, with well-being (of any definition) being the aim of the service or research or linked to the reported outcomes. As we wanted to explore how rehabilitation can affect well-being, we included studies that reported mostly negative impacts on well-being. We included studies in which participants discussed a rehabilitation service (as defined above), including services provided outside of an inpatient rehabilitation setting or conducted by non-professionals. One area of contention was whether adapted sport should be considered a rehabilitation service: we included studies that involved entry-level adapted sports, but not elite sports. Included studies needed to have employed a qualitative methodology. Qualitative methods needed to provide an in-depth understanding of the perspective of people with SCI, so we excluded studies that only used closed-ended questionnaires or surveys. Mixed-methods studies were included if the qualitative component facilitated this in-depth understanding. We also included reviews of qualitative studies. We included studies published between 1995 and May 2021, assuming that research published more than 25 years ago would reflect a different service delivery context and that results of such studies would only be minimally useful to our research aims. We only included studies published in English, for pragmatic reasons.

The authors needed to include participant quotations as an ‘audit trail’ to support their findings and to ensure the voices of people with SCI were represented. This was the only indicator of methodological quality that was used as a basis for inclusion. Quality of included studies was further appraised using the Critical Appraisal Skills Programme (CASP) qualitative checklist (Skills, C.A. and Programme, 2018). This checklist was used to evaluate and report the methodological quality of the included studies, but was not used to determine inclusion or exclusion. We only appraised the qualitative component of mixed-methods studies and only appraised primary research using this checklist.

**Figure 1.**
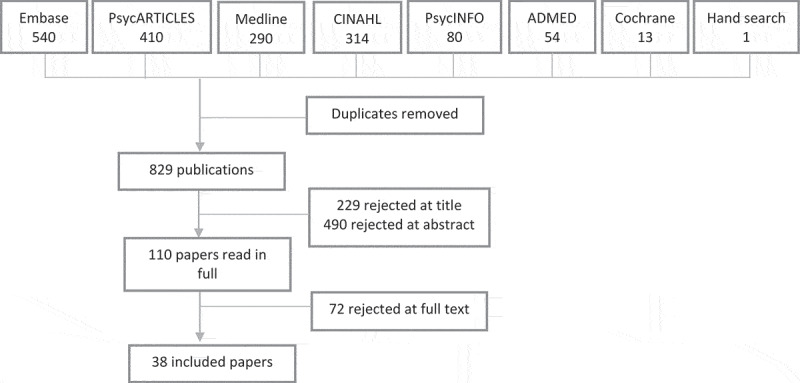
Flowchart of study selection

Study titles, abstracts and then full-text versions were screened for relevance, and we gradually excluded studies that were not relevant or did not meet the inclusion criteria. The titles of 829 original publications were screened, with 229 studies excluded at this stage. The abstracts of 600 studies were read, where the title suggested relevance or did not provide enough information. After 490 studies were excluded based on their abstract, 110 studies were read in full. Of these, 72 studies were excluded, including 8 studies that otherwise met the inclusion criteria but did not meet the quality criterion of including participant quotes. Other reasons for exclusion included study participants not receiving a common intervention or the study having an inadequate or unclear focus on well-being. A flow chart of study selection is shown in Figure 1.

## Stage 4 and 5: Charting the data, and collating and summarizing the results

We developed charting forms related to each research aim and extracted the following data: study location, methods and sample; conceptions of well-being; program type, timing, duration and context; specific program activities; and valued aspects, limitations and perceived outcomes of programs. Next, we used reflexive thematic analysis to identify themes, using the methods described by Braun & Clarke (Braun & Clarke, 2019, 2012, 2014). We coded findings that appeared to relate to a similar category, using an iterative coding strategy. For example, we grouped findings about the characteristics of program staff and separately grouped findings about the involvement of peers with SCI. These categorized findings functioned as ‘topic summaries’ which were then analysed inductively and interpreted to identify patterns of shared meaning in the perspectives of the study participants (Braun & Clarke, 2021). The first author (BS) conducted the analysis, with team discussions used for reflexivity and to provide additional perspectives to guide coding, interpretation and theme development.

## Results

### Research aim i): examine the extent and nature of qualitative research related to well-being programs for people with SCI

Study characteristics are described in Table I. Of the 38 included studies, 28 used a solely qualitative methodology, one was a systematic review of qualitative studies, seven reported both qualitative and quantitative results of a mixed-methods study, and two reported only the qualitative results of a broader mixed-methods study. The quantitative component of mixed-methods studies mostly involved a single cohort pre-post design (n = 5), and only one mixed-methods study involved a randomized controlled trial.

**Table I.** Study characteristics

Study	Location	Aim	Methods	Sample	Intervention	Conception of well-being
Beauchamp et al., (2016)	Canada	Explore the perspectives of mentees with SCI about peer mentoring (in reference to transformational leadership concepts)	Semi-structured individual interviews	15 people with SCI Aged 25–69 years 14.5 (SD 16) years post-injury	Peer mentoring	Background included literature about link between peer mentoring, transformation leadership, well-being and life satisfaction. Improved ‘overall well-being’ was a finding but not defined.
Bernet et al., (2019)	Switzer-land	Evaluate patient perspectives and experiences of a nurse-guided education program	Semi-structured individual interviews- shortly before discharge and 5–6 months post-discharge	10 people with SCI Aged 19–67 years L2-C3 injury	Nurse-led individualized education program in inpatient setting	Improved well-being was a finding, but not defined.
Block et al., (2010)	USA	Evaluate outcomes of a capacity-building program, in terms of self-efficacy, ability to set and achieve goals, and independent living status	Mixed-methods: Non-randomized controlled trial Semi-structured individual interviews	35 people with long-term neurological impairment in broader study (16 with SCI), 19 were interviewed (10 with SCI) Mean age 44	Community-based capacity-building program Control groups: wait list or no intervention	Background included literature about link between well-being, goal setting ability and self-efficacy.
Brillhart & Johnson, (1997)	USA	Explore experiences in rehabilitation (particularly nursing interventions), which affected coping, from the perspective of people with SCI	Individual interviews	12 people with SCI 6 with paraplegia, 6 with tetraplegia Aged 18–77 10 (2–21) years post-injury	Inpatient SCI rehabilitation	Background included literature about contribution of effective coping to QoL.
Chemtob et al., (2018)	Canada	Explore perceptions of mentees with SCI about peer mentoring (in reference to self-determination theory)	Semi-structured telephone interviews	13 people with SCI Mean age 49.3 years 54 per cent with paraplegia 15.3 years post-injury	Peer mentoring	Background included literature about link between peer mentoring and QoL.
Conti, Dimonte et al., (2020)	Italy	Identify barriers and facilitators to education provided during SCI rehabilitation.	Focus group interviews	22 people with SCI Mean age 49 years 14 with paraplegia, 8 with tetraplegia 4.5 years post-discharge	Education provided throughout rehabilitation journey	Self-management programs aim to improve QoL. Background discussed need to identify how these programs can better increase well-being.
Cotner et al., (2018)	USA	Examine quality of life for participants of a vocational program	Mixed-methods: Pre-test/post-test design. Face-to-face semi-structured interviews	151 people with SCI interviewed (from 213 in quantitative sample) Mean age 51 years	Individual Placement and Support (IPS) aiming to secure employment in the open market	Well-being linked to WHO definition of health (World Health Organization, 2006). Background included literature about contribution of employment to QoL.
Ekelman et al., (2017)	USA	Understand the experiences of participants of a fitness program for people with SCI and perceived influences on well-being, (particularly in relation to occupational science concepts)	Semi-structured individual interviews, observation	4 men with SCI Aged 26–49 years C5-C3 tetraplegia 7.4 (1–22) years post-injury	Accessible fitness program at a community wellness centre	As defined by Wilcock, (2006): Physical well-being (health status and beliefs, and ability to perform valued activities); mental well-being (positive mood, self-esteem, coping, problem-solving, decision-making, meaning); and social well-being (relationships and making contributions). Well-being linked to occupations and physical activity.
Folan et al., (2015)	Australia	Understand the experiences of clients trialling assistive technology for computer access during rehabilitation	Semi-structured individual interviews	7 men with tetraplegia (C4–5). Aged 20–60 years 21 (6–29) months post-injury	Inpatient trial and practise of assistive technology (AT) for computer access	Discussion included literature about contribution of computer access and engagement in meaningful occupations to QoL. A finding was improved self-efficacy “therefore improved well-being”.
Hall et al., (2021)	USA	Explore the experience of people with SCI, to inform a new rehabilitation continuum of care.	Semi-structured individual interviews	10 people with SCI Aged 29–65 years 1–20 years post-SCI		Described various factors affecting quality of life, including determinants identified by Hammell (2007). Discussion included need for rehabilitation to focus more on wellness.
Hitzig et al., (2013)	Canada	Compare the effects of a FES-assisted mobility intervention with a non-FES exercise program, in relation to QoL and participation	Mixed methods: Parallel group RCT. Individual interviews with participants from both groups	16 people with SCI interviewed (from 34 in quantitative sample). C2-T12 (incomplete) Mean age approx. 55 years 8.75 (8–10) years post-SCI	Treatment: Functional Electrical Stimulation (FES) and mobility training with physiotherapist Control: resistance and aerobic training with kinesiologist	QoL was a key outcome of interest, but not defined. QoL, self-confidence and community participation, positive mood, self-confidence, and self-efficacy were stated as domains of well-being. QoL and well-being often used interchangeably. Improved well-being was a finding.
Houlihan et al., (2003)	USA	Explore the self-reported benefits of internet use for people with SCI, impacts on social participation and health-related QoL, and perceived risks	Mixed methods: Pilot pre-test/ post-test study Monthly semi-structured telephone interviews	23 people with SCI living in the community. Aged 18–63 years	Provision of internet access and hardware	Health-related QoL was primary outcome of interest, but not defined. ‘Improved QoL’ was a key finding/theme. Discussion included contribution of leisure activities to life satisfaction.
Hutchinson et al., (2003)	USA	Explore how people use leisure as a coping resource after a traumatic injury or chronic illness	Semi-structured individual interviews (face-to-face or telephone)	Qualitative data from two broader studies, interviews with 16 people with disabilities (12 with SCI) Aged 24–63 years 7 with tetraplegia, 5 with paraplegia	Engagement in leisure activities (including involvement of therapeutic recreation services)	Main focus of this paper was coping, the description of which shared similar domains to WB. An aim of the broader study was to explore subjective WB (not defined). Discussion linked coping to positive psychology.
Labbé et al., (2019)	Canada	Explore the benefits of adaptive recreational leisure activities to health and social participation, (particularly quality of participation), and barriers and facilitators to participation.	This paper reports the qualitative results of a mixed-methods study. Semi-structured individual interviews, focus groups, and observation	19 people with disabilities (4 with SCI), 9 volunteers and 8 staff members of a community-based recreation program Mean age 48.5 years	Adapted recreational leisure activities (RLA) program	Low social participation adversely affects WB. RLA may target this issue. Quality of participation was key topic of interest (conceptualized by Ginis et al as autonomy, belonging, challenge, engagement, mastery and meaning); thought to have more impact on WB than frequency of activities. Choice and control linked to WB.
Lai et al., (2016)	USA	Explore the feasibility, potential effects (including well-being) and acceptability of a tele-exercise program for people with SCI	Mixed methods: single cohort pre-post study, Individual interviews	4 people with SCI Mean age 43.5 years T1–2, T2–3, T10–11 and C4–5 25.8 (20–30) years post-injury	Tele-exercise program	Subjective well-being an outcome of interest, but not defined.
Lape et al., (2018)	USA	Identify factors that influence participation in community-based adaptive sport programs	Focus groups	17 people with disabilities (including 4 with SCI, 10 wheelchair users). Aged 21–63 years Median 22 (5–52) years post-injury/diagnosis	Community-based adaptive sports program	Background included literature about contribution of adaptive sports to life satisfaction. Findings included benefits to physical well-being.
Luchauer & Shurtleff, (2015)	USA	Identify meaningful components of exercise and adaptive recreation for people with SCI, and explore relationships to performance, capacity and participation	Focus groups and individual interviews	17 people with SCI. Age in 20s to 50s. 7 with paraplegia, 10 with tetraplegia 11.7 (1.5–37) years post-injury	Existing involvement in regular physical activity (PA) through local organizations	Background included literature about contribution of improved capacity and performance (as defined in ICF) to QoL.
Lucke, (1997)	USA	Describe the process, meaning and consequences of nurse caring during rehabilitation from the perspective of people with SCI	Semi-structured individual interviews throughout rehabilitation	15 people with SCI Mean age 48 years 8 with paraplegia, 7 with tetraplegia	Nursing care interventions at two SCI rehabilitation centres	A desired implication of the study is to design interventions to improve problem-solving, well-being and QoL. Improved well-being was a finding, but not defined.
Maddick, (2011)	Australia	Evaluate a music therapy program, including participant and practitioner experiences	Semi-structured individual Interviews face-to-face, or telephone. Focus group with practitioners	13 men with SCI Aged 17–59 years 9 with tetraplegia	Music therapy with music therapist and social worker during inpatient rehabilitation	Introduction includes themes of quality of life for people with SCI described by Manns & Chad, (2001): physical function and independence, physical accessibility, stigma, emotional well-being, relationships and social function, spontaneity, occupation, finances and physical well-being.
Mattar et al., (2015)	Canada	Understand how people with SCI use information technology (IT), and ways IT may be used to support health and well-being	Individual, semi-structured interviews Data also collected about quality of life (WHO-QoL BREF) and self-efficacy (Moorong Self-Efficacy Scale)	10 people with SCI Aged 20–75 years 6 with paraplegia, 2 complete and 2 incomplete tetraplegia. 10.5 (2.5–26) years post-injury	Existing use of IT and specialized access equipment/software	Well-being (physical, mental and social) was the topic of interest; not defined. Background includes research about benefits of IT to well-being through access to telehealth services, on-line resources, and peer support.
Nygren-Bonnier et al., (2018)	Sweden	Describe and explore the experiences of people with tetraplegia learning and using glossopharyngeal breathing	Semi-structured telephone interviews. Participants of an earlier intervention study (Nygren-Bonnier et al., 2009)	26 people with tetraplegia Mean age 47 years C8-C4 21 (2–51) years post-injury	Training in glossopharyngeal breathing by a physiotherapist	Improved well-being was a finding, related to physiological improvements, positive emotions and reduced stress and anxiety. Discussion included literature about contribution of autonomy to quality of life.
O’Dell et al., 2019)	UK	Evaluate a peer support program, and its role in multidisciplinary support for people with SCI	On-line survey, semi-structured telephone interviews, and focus groups	100 people with SCI, their family and friends, peer support officers.	Peer support program for people with SCI, family, friends and healthcare providers	Background included literature about contribution of social support to QoL.
Ramakrishnan et al., (2016)	Australia	Explore experiences and perceptions of people with SCI of an early intervention vocational program	Semi-structured individual interviews	13 people with SCI Aged 19–60 years L4-C3 14 (7–21) months post-injury	Pilot early intervention vocational rehab (VR) program	Background included literature about contribution of employment to QoL and subjective well-being.
Semerjian et al., 2005)	USA	Assess the effects of adapted exercise on quality of life and body satisfaction of people with SCI	Mixed methods: Single cohort pre-post study, field observations, semi-structured interviews	12 people with SCI Mean age 34 years T5-C5 6 (1–30) years post-injury	Adapted exercise program	QoL defined as “an individual’s assessment of their level of satisfaction in several components of their lives” [p96]; this subjective assessment as defined by Noreau & Shepherd as “the gap between an individual’s aspirations and current achievements” (Noreau & Shephard, 1995) p229
Singh, Shah et al., (2018)	Canada	Understand perceived impacts of a mobility training intervention on the lives of people with SCI, and their experiences of the intervention	Semi-structured interviews	7 people with SCI Mean age 57.3 years T8-C4 incomplete injury 4 (3–6) months post-injury	Intensive outpatient locomotor training conducted by a physiotherapist	Findings were summarized as improvements to physical and psychological well-being.
Singh, Sam et al., (2018)	Canada	Long-term follow-up from above study: Explore perceived long-term effects on function and community living	Semi-structured telephone interviews Follow-up interviews 1–2 years after earlier study (above)	6 people with SCI Aged 49–65 years T8-C4 incomplete injury 2 (1.9–2.7) years post-injury	As above	Some findings (changes to mood and sense of self) were reported as ‘changes in psychological well-being’.
(Swaffield et al., (2021)	Canada	Explore perceptions of people with SCI about activity based therapy (ABT)	Semi-structured interviews	Ten people with SCI Median age 28.5 years 6 with tetraplegia, 4 with paraplegia Median 7.3 (2.5–23) years post-SCI	Community-based ABT targeting motor and sensory function	Background included literature about contribution of ABT use to QoL. Improved well-being and QoL reported in results.
Tamplin et al., (2014)	Australia	Explore participant experiences of group music therapy	Qualitative results of a mixed-methods study. Semi-structured individual interviews	24 people with SCI Aged 27–70 years (mean 45) T1-C4 Median 9 (1–26) years post-injury	Treatment: Group singing and respiratory training Control: Group music appreciation and relaxation	Improved well-being was a finding, but not defined. Linked to socialization and physical activity. Discussion about contribution of music to flourishing.
Taylor & McGruder, (1996)	USA	Identify meaningful components of sea kayaking and examine processes that may underlie perceived positive changes	Individual ethnographic interviews, observation	3 people with incomplete SCI around C6: non-ambulatory with some UL function. Aged 23–38 years 5 (3–10) years post-injury	Sea kayaking expedition led by a recreational therapist	Background included literature about link between life satisfaction/QoL and engagement in activities, particularly those related to leisure and physical activity.
Veith et al., (2006)	USA	Explore peer mentoring from the perspective of mentees with SCI, including areas of adjustment and the mentoring relationship	Individual telephone interviews	7 people with SCI Mean age 40 kayaking 5 paraplegic, 2 tetraplegic	Peer mentoring program during inpatient rehabilitation	Background included literature about contribution of social support to QoL/well-being.
(Verdonck et al., (2011)	Ireland	Explore contribution of an environmental control system (ECS) to participation in everyday life	Focus groups	15 people with high level tetraplegia (C3-C5) 20–57 years old. 10 (1–31) years since discharge from inpatient rehab	Existing or imagined use of an ECS	Background included literature about contribution of ECS use to QoL. Discussion linked the themes of this study (and earlier research) to various elements of well-being and quality of life.
(Verdonck et al., (2014)	Ireland	Explore user perspectives of ECS, and the potential of ECS in mitigating participation restrictions and activity limitations	In-depth, individual interviews	6 people with high level tetraplegia (C3-C5). Aged 22–65 years 13 (3–35) years post-discharge	8 week loan of a customized ECS ‘starter pack’ enabling control of home appliances	Background included literature about contribution of ECS use to QoL.
Verdonck et al., (2018)	Ireland	As above (Verdonck et al., 2014): This study reports additional findings	As above	5 people with high level tetraplegia (C3-C5). Aged 22–55 years At least 3 years post-discharge	As above	As above. Discussion included literature about contribution of doing everyday things to quality of life.
Wangdell et al., (2013)	Sweden	Explore effect of reconstructive hand surgery on everyday life	Semi-structured individual interviews, 7–12 months post-surgery	11 people with tetraplegia (C4-C7) Aged 22–73 years 3 (2–6) years post-injury	Reconstructive hand surgery to improve grasp	Introduction: Improved QoL would be an expected outcome of improved hand function. Discussion: Findings were summarized as improved self-efficacy, which is linked to QoL.
Ward et al., (2007)	USA	Explore the social and occupational participation of people with SCI, and perceptions about occupation-based interventions in achieving these outcomes	Semi-structured individual interviews	3 people with SCI 2 paraplegic, 1 tetraplegic 2–5 years post-injury	Occupation-based occupational therapy interventions (inpatient and community settings)	Introduction included literature about link between maintenance of daily activities and life satisfaction.
Wellard & Rushton, (2002)	Australia	Explore the perceptions of people with SCI about nursing practises for pressure ulcer management, particularly in relation to spatial practises and environment	In-depth, unstructured interviews	15 people with SCI Family members also involved in 8 of the interviews	Nursing care for pressure ulcer management in inpatient SCI service	A major finding was the influence of spatial practises on physical, emotional and social well-being (not defined). Mostly these had a negative impact on well-being.
Williams et al., (2014)	UK	Synthesize qualitative research about leisure-time physical activity (LTPA) for people with SCI, including, and propose improvements to LTPA promotion	Systematic review of 18 qualitative studies about LTPA for people with SCI, from 2000–2012	Community-dwelling people with SCI	LTPA programs	Well-being defined as “optimal physiological function and experience” including subjective WB (SWB) (life satisfaction and happiness), psychological WB (PWB) (psychological growth and development; and social WB- flourishing and function in social life).
Zinman et al., (2014)	Canada	Evaluate the effectiveness of a community reintegration program for promoting well-being and community participation post-SCI	Mixed-methods study: single cohort pre-post study. Semi-structured individual interviews with 12 of the participants	21 people with SCI. Mean age 46 years 3.6 years post-injury 12 tetraplegic	Self-management program, facilitated by OTs and social workers	Hypothesis was the program would improve psychological, emotional and social WB, but these terms were not defined. Discussion included literature about contribution of coping strategies to QoL.

Four of the studies recruited people with other conditions, in addition to people with SCI. These other conditions appeared as all cause long-term physical disability. Eight studies exclusively recruited people with tetraplegia; the other studies had a mix of people with paraplegia and tetraplegia (n = 20), or did not report level of injury (n = 9). Mean time post-injury in the studies was less than one year (n = 1), 1–2 years (n = 3), 3–5 years (n = 5), 6–10 years (n = 7), 11–15 years (n = 5), more than 16 years (n = 3) or was not stated (n = 13). All the studies involved adults, and no paediatric studies were found. Three of the studies included interviews with family members and health professionals in addition to people with SCI. The majority of studies were conducted in USA (n = 14) and Canada (n = 9), with other studies conducted in Australia (n = 5), Ireland (n = 3), Sweden (n = 2), UK (n = 2), Italy (n = 1) and Switzerland (n = 1).

Quality appraisal findings are reported in the supplementary material. The most common methodological issues were methods (e.g., interview guide) not being made explicit, no discussion of data saturation, and a lack of reflection of how researcher biases may have influenced design, recruitment and analysis. Most studies did not report ethical, methodological or recruitment issues, which may have been because such issues did not arise.

### Research aim ii): describe how well-being is conceptualized in these studies, and whether/how intentional design for well-being was used

The term ‘well-being’ was used in 16 of the included studies, but the most commonly used term was quality of life (n = 21). Other terms used that appeared to relate to well-being included life satisfaction (n = 5), social well-being (n = 5), physical well-being (n = 4), psychological well-being (n = 4), subjective well-being (n = 3), mental well-being (n = 2), emotional well-being (n = 2), flourishing (n = 2), health-related quality of life (n = 1), psychosocial well-being (n = 1) and overall well-being (n = 1). However, in the majority (n = 31) of studies, these terms were not defined. Some authors used multiple terms (e.g., ‘well-being’ and ‘quality of life’) and these mostly appeared to be used interchangeably.

Of the studies that defined well-being (or quality of life), four listed a broad range of well-being elements, relating to physical functioning, psychological/emotional resources and state, social functioning, independence and participation, and environmental accessibility (Ekelman et al., 2017; Hitzig et al., 2013; Maddick, 2011; Williams et al., 2014). One study defined quality of life as the gap between desired and actual achievements (Semerjian et al., 2005). Another defined quality of life using the World Health Organization’s broad definition of health (Cotner et al., 2018).

Some studies focused on a specific phenomenon they linked to well-being, including the following: occupations and meaningful activities (Ekelman et al., 2017; Folan et al., 2015; Luchauer & Shurtleff, 2015; Verdonck et al., 2018; Ward et al., 2007), physical activity (Ekelman et al., 2017; Taylor & McGruder, 1996), adaptive sports (Lape et al., 2018), leisure activities (Houlihan et al., 2003; Labbé et al., 2019; Taylor & McGruder, 1996), peer mentoring (Beauchamp et al., 2016; Chemtob et al., 2018), goal-setting ability and self-efficacy (Block et al., 2010; Folan et al., 2015; Wangdell et al., 2013), employment (Cotner et al., 2018; Ramakrishnan et al., 2016), computer/IT access (Folan et al., 2015; Mattar et al., 2015), coping (Brillhart & Johnson, 1997; Hutchinson et al., 2003; Zinman et al., 2014), choice and control (Labbé et al., 2019), autonomy (Nygren-Bonnier et al., 2018), social support (O’Dell et al., 2019; Veith et al., 2006), social participation (Tamplin et al., 2014), music (Tamplin et al., 2014), and use of environmental control systems (Verdonck et al., 2014, 2011).

### Research aim iii): describe specific activities, timing and context of rehabilitation services related to well-being

#### Activities

**Figure 2.**
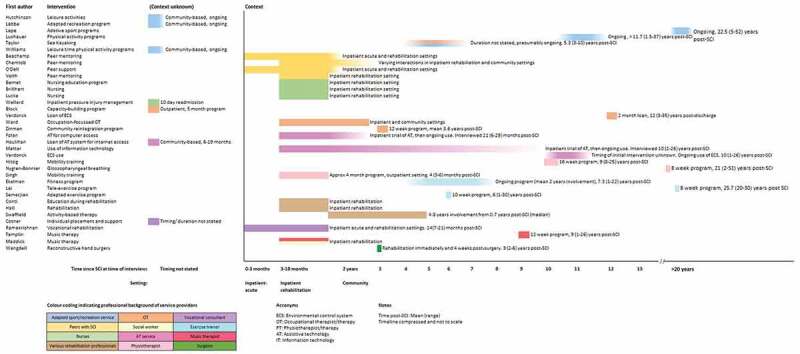
Service timing and context

Service activities are described in Table II, and the timing and context of services are shown in Figure 2. A broad range of service types were studied, carried out by a range of disciplines. We categorized the services based on who delivered them: adaptive recreation and sport providers (n = 6), peers with SCI (n = 4), nurses (n = 4), occupational therapists (n = 4), an occupational therapist and social worker (n = 1), assistive technology services (n = 4), physiotherapists (n = 4), exercise trainers (n = 3), vocational consultants (n = 2), various rehabilitation professionals (n = 2), a music therapist (n = 1), a music therapist and social worker (n = 1), and a surgeon (n = 1). Most services were delivered by a single profession, although presumably some of these services were part of a broader multidisciplinary program. Two studies described a multidisciplinary service.

**Table II.** Service activities

Delivered by	Authors	Intervention type	Intervention description
Adapted recreation/ sports service	Hutchinson et al., (2003)	Adapted recreational leisure activities	(Six participants with disabilities) therapeutic recreation program in a rehabilitation hospital, with individual and group therapy (Ten people with SCI) leisure engagement: intervention (if any) not described
	Labbé et al., (2019)	Community-based adapted recreational leisure activities (RLA)	Community-based RLA program for people with disabilities, run by a non-profit organization, in community/council facilities and a rehabilitation centre Programs include sailing, paddling, hiking, gardening, wood crafting, music, creating assistive technology, social/information gatherings
	Lape et al., (2018)	Adapted sports	Community-based adapted sports program, affiliated with a rehabilitation hospital network Sports include cycling, sailing, rowing, golf, yoga, kayaking, dance, Nordic skiing
	Luchauer & Shurtleff, (2015)	Regular physical activity—adapted recreation	Local community organizations providing accessible physical activity
	Taylor & McGruder, (1996)	Sea kayaking expedition with recreational therapist	Sea kayaking through an outdoor experience organization for people with disabilities In-pool training, including rescue and water exit drills, Sea kayaking expeditions with recreation therapist Preparatory contribution of OTs in analysing problems and adapting seatings systems and paddles.
	Williams et al., (2014)	Leisure time physical activity	Physical activities engaged in during spare time, e.g., recreational sport, gym exercise
Peers with SCI	Beauchamp et al., (2016)	Peer mentoring program	Formal peer mentoring program of two NGOs Trained peer mentors provide information and support related to living with SCI Support ranged from 1–2 meetings, to more than a year
	Chemtob et al., (2018)	Peer mentoring program	Peer mentoring program of a provincial organization Mentoring provided by employees with basic training, in inpatient and/or community settings Mentoring activities included conversations about living with SCI, family member discussions, resource provision and outings Informal mentoring at events run by the organization
	O’Dell et al., (2019)	Peer support program	Peer support program of a spinal injury association Training by peer support workers for healthcare practitioners
	Veith et al., (2006)	Peer mentoring program	Peer support program of inpatient rehabilitation unit Matching of trained peer mentors to mentees (similar injury level, gender and age) 1–5 face-to-face meetings
Nurses	Bernet et al., (2019)	Nurse-led inpatient education program	Assessment, collaborative goal setting/review and joint development of structured program Education program (focused on attainment and application of knowledge and skills, gradual increase in responsibility) involving written information, seminars and workshops, application of skills to practical tasks Peer counselling
	Brillhart & Johnson, (1997)	Inpatient rehabilitation, particularly nursing	Inpatient rehabilitation (not described in detail) with provision of nursing care Education and skills training
	Lucke, (1997)	Nursing care during inpatient rehabilitation	Provision of nursing care during inpatient rehabilitation (not described in detail)
	Wellard & Rushton, (2002)	Inpatient pressure ulcer management	Inpatient admission from the community and treatment of pressure injury Focus was the type and use of space in which nursing care was provided, including the way staff adjusted the environment to influence an activity, as well as impacts of the existing spatial arrangements on practise
Occupational therapist	Block et al., (2010)	Capacity- building program	10 group meetings over 5 months Morning seminars: Lectures, group discussion and activities, role play Topics (tailored to participant interests) included communication, self-advocacy, adaptive equipment and health promotion Individual goal-setting; provision of strategies (e.g., information about local resources) and peer support for attainment Afternoon: indoor and outdoor physical or recreational group activities, in various community-based settings Case co-ordination and peer counselling through a partner organization Described further in a companion study (Block et al., 2005)
	Verdonck et al., (2014 and Verdonck et al., (2018)	Loan of environmental control system (ECS)	8 week loan of a customized ECS ‘starter pack’ ECS enabled control of telephones, lamps, fans, AV equipment and a personal alarm, via a switch-operated remote control Customization, assembly and training provided by an OT researcher
	Ward et al., (2007)	Occupation-based OT interventions	Occupation-based OT interventions (assessment, goal-setting and intervention focused on valued occupations) Inpatient (n = 2) and community rehabilitation (n = 1) settings Intervention activities included practising valued occupations, e.g.,, shopping and cooking, skills training, escorted outings and facilitated family outings, environmental modifications linking with community-based organizations
Occupational therapist and social worker	Zinman et al., (2014)	Self-management education program	Weekly, 12-week education program facilitated by an OT and social worker at an SCI rehabilitation hospital. Focus on self-efficacy, self-management, community integration and well-being Education activities included lectures, reflection, group discussion and activities, written information, homework tasks to reflect on and apply learning Topics included self-care, adjustment, stress management, problem-solving, emotions, self talk, communication, energy and pain management, and well-being Individual goal-setting, and group monitoring/facilitation of goal attainment Community outing Guiding principles were cognitive behavioural therapy, adult learning, goal-setting, client-centred care, Canadian Model of Occupational Performance.
Assistive technology service (background not specified)	Folan et al., (2015)	Assistive technology (AT) for computer access	Exposure to AT for computer access as part of an SCI rehabilitation service (clinician role not described) AT included speech recognition software, trackball and mouth joystick devices, finger splints. Computer tasks include internet browsing, social media, letters and email, online books, banking, shopping, school work, work tasks and games.
	Houlihan et al., (2003)	Internet access	Free internet access for 6–19 months WebTV hardware—TV monitor and wireless keyboard Installation, basic instruction, technical assistance by researchers and product support hotline
	Mattar et al., (2015)	Existing use of IT and specialist access equipment	Some participants had input from assistive technology departments in SCI rehabilitation Involvement of staff not otherwise described, and some of the problems identified suggest a lack of expert involvement
	Verdonck et al. (2011)	Existing or imagined use of environmental control system	Specific intervention activities not described. ECS systems included specialized environmental control units and one mainstream home control system (X-10)
Physio-therapist	Hitzig et al., (2013)	Functional electrical stimulation (FES) and treadmill walking/ exercise training	Mobilization on a body-weight supported treadmill. Graded support of body weight using a harness. FES stimulation to both legs, manually triggered by a physiotherapist or (rarely) the participant. Manual assistance by up to 3 assistants if needed, to facilitate walking pattern Control group—exercise program with resistance and aerobic training supervised by kinesiologists.
	Nygren-Bonnier et al., (2018)	Glosso-pharyngeal breathing training	Training in glossopharyngeal breathing by a physiotherapist The 8-week intervention, as described in a companion paper (Johansson et al., 2011), involved a training video, written information and supervised practise of the technique.
	Geard et al., (2018) and Bergmark et al., (2008)	Mobility training program	Four 90-minute sessions/week continued until progress plateaued. Program began within 4 weeks of discharge from inpatient rehabilitation Step training on body weight supported treadmill, overground walking
Exercise trainer	Ekelman et al., (2017)	Community fitness centre	Community fitness centre designed for people with SCI 1:1 personal training Accessible and specialized equipment, e.g.,, body-weight supported treadmill, FES exercise machine, standing frame
	Lai et al., (2016)	Teleexercise program	Eight-week, 3x/week exercise program using upper body ergometer Real-time coaching and monitoring by an exercise trainer via tablet computer and biometric monitors Written instructions, visual targets for each session Initial setup conducted in-person
	Semerjian et al., (2005)	Adapted exercise program	Ten-week individualized exercise program Wheelchair-accessible weight machines, arm and leg ergometer (active and/or passive movement), standing frame with passive leg movement controlled by active arm movement, and body weight supported treadmill
Various rehabilitation professionals	Conti, Dimonte et al., (2020)	Education	Skill training and education Home visits, overnight leave, community outings
	Hall et al., (2021)	Rehabilitation	This study reported mostly limitations in specific rehabilitation activities Peer groups and support Most positive reports related to support of significant others, and own psychological resources
	Swaffield et al., (2021)	Activity based therapy	Functional electrical stimulation, task-specific practise, weight-bearing exercises, locomotor training. Can be delivered by occupational therapists, physiotherapists, kinesiologists and rehabilitation assistants.
Vocational consultant	Cotner et al., (2018)	Individual Placement and Support for employment	Individualized job support, e.g.,, workplace accommodations Liaison with clinical team to foster integration vocational goals into general rehabilitation, ongoing support after employment
	Ramakrishnan et al., (2016)	Early intervention vocational program	Vocational rehabilitation program provided by a vocational consultant in acute and rehabilitation setting Vocational assessment and intervention Collaboration with clinical team, integration of vocational rehabilitation into general rehabilitation program
Music therapist and social worker	Maddick, (2011)	Music therapy program	Individual sessions with a music therapist, including song writing, relaxation, singing, voice therapy, playing instruments Weekly group sessions with social worker and music therapist: song writing, relaxation, and music discussions. Social worker facilitated group processes and peer support. Can be delivered in various settings. This study focused on community-based delivery.
Music therapist	Tamplin et al., (2014)	Group singing/music appreciation	Treatment: 12 weeks of active music therapy, involving group singing using Neurologic Music Therapy Techniques, respiratory and vocal exercises. Control: group receptive music therapy involving music appreciation and discussion, musical games and relaxation Both interventions conducted in an outpatient setting and facilitated by a music therapist
Surgeon	Wangdell et al., (2013)	Reconstructive hand surgery	Reconstructive surgery to restore grasp 5 days of rehabilitation immediately and 4 weeks after surgery

Services were often only described in general or vague terms, although mixed-methods studies tended to include a more detailed description of specific intervention activities. Whilst a range of disciplines delivered the services, we have identified and categorized activities that were common to the services, including the following: structured education programs (e.g., workshops), facilitating engagement in occupations and activities (e.g., skills training, adapting activities, escorted outings, and group activities), facilitating access to assistive technology (e.g., exposure, prescription, loan, training, and modifications), psychological and emotional support (e.g., coaching, training, goal setting, goal pursuit, and support groups), formal and incidental peer support and mentoring, addressing body function (e.g., mobility training, breathing training, and electrical stimulation), liaison (e.g., integrating program into general rehabilitation, and referral to other organizations), nursing care and surgery.

### Research aim iv): explore how people with SCI perceive and experience these services

Valued aspects, limitations and perceived outcomes of the services are described in Table III.

**Table III.** Valued aspects, limitations and perceived outcomes

Authors, service type	Valued aspects	Limitations	Perceived outcomes
Beauchamp et al., (2016) Peer mentoring program	Motivation: encouragement, realistic optimism, high expectations Role modelling: trust, setting an example, setting expectations Caring behaviours: empathy, understanding, individualized support Empowering: advice, problem-solving strategies, reframing problems	Not reported	Increased motivation, hope, self-confidence, acceptance, “overall well-being” Increased social participation
Bernet et al., (2019) Nurse-led inpatient education program	Information about relevant and real-life situations, and written information for later use Practical application of knowledge/practise of skills (including opportunity to experiment and practise alone) Goal-setting and collaboration Opportunities for discussions with peers Flexibility in provision, e.g.,, around rest periods Interpersonal skills of staff: understanding, individualized care, motivation	Need more ‘mental preparation’ for challenges when returning home: new realization of limitations, more time and space to think, and environmental barriers outside of ‘ideal’ ward environment	Self-confidence Improved skills and capability
Block et al., (2010) Capacity- building program	Support for goal attainment Information about rights, and increased recognition of the need for self-advocacy Role-playing provided opportunities to practise skills, e.g.,, self-advocacy Peer support—advice, sharing concerns, positive examples/success stories Changed perceptions about their potential Increased awareness of accessible activities and community resources Increased awareness of importance of environment (vs impairments) to participation Learning a new mindset towards problems, problem-solving strategies Program attendance provided opportunities for socialization and new friendships	Social, financial and access barriers still limited participation/goal attainment for some participants Barriers to self-advocacy still existed, e.g.,, perspective not being listened to by health professionals, discomfort in doing so	Improved community access and participation Attainment of independent living goals Engagement in, or working towards, new occupations, e.g.,, paid and voluntary work, education, recreation activities Improved physical activity, weight loss, decreased medication Decreased isolation
Brillhart & Johnson, (1997) Inpatient rehabilitation—particularly nursing	Nurses taking every opportunity to teach skills Influence of peers: role models, positive examples, problem-solving, resources. Having the opportunity to contribute to others in the same way Being treated as an individual and with dignity: being listened to, warm interactions, ‘homey’ environment and casual dress of staff, being treated as a ‘regular average person’, staff spending time with them, having their personal appearance attended to, matter-of-fact attitudes of nurses during personal care procedures. Having their own expertise respected and encouraged: assessing and solving own problems Continuity/consistency of staffing Pursuing long-term goals and resuming previous activities Positive expectations of others about potential (including family members) Elimination of environmental barriers, access to resources to promote independence Other facilitators included own problem-solving and support of significant others, having important roles.	Feeling reluctant to leave perceived safety of the rehabilitation setting Platitudes Questions being discouraged Being provided with unnecessary assistance Fear of risks (e.g., falling) and use of analgesic medication limited independence	Self-esteem, confidence, continued/resumed sense of self Accomplishment of important tasks and participating in valued roles Realization life isn’t over, “*I still had all these opportunities to live yet*”. (p. 253) Perspective about, and adjustment to, situation (often took 1–3 years) Positive attitude about self, which influences attitudes of others
Chemtob et al., (2018) Peer mentoring program	Being involved in the decision-making process, feeling in control of the mentoring sessions Content and style of sessions tailored to individual needs and personality Being able to ask questions and be listened to Flexibility in session timing, mentors approachable and available Care, empathy, comfort, reassurance, friendship Sensitivity and understanding from shared experience Role modelling, an example of what is possible and methods for achievement Realism about situation, problems and prognosis Advice and reassurance provided to family members	Goals not being supported, or actively discouraged Not feeling understood Events and activities too far away or too late at night	Autonomy, competence Positive expectations Emotional benefits Expanded networks
Conti, Dimonte et al., (2020) Education provided during rehabilitation	Self-management strategies helped with motivation and applying education Able to engage in education programs once they accepted a long-term change in their life Goal setting and planning to pace and prioritize education Own psychological strengths, especially motivation and determination Support of family in overcoming barriers Peer support and interaction Home visits and overnight leave in preparation for discharge Opportunities to engage in community and leisure activities whilst in hospital (rare)	Not ready to receive, value or understand information in early stages Lack of energy to learn and apply information Need more time to learn skills and information, including beyond inpatient admission. Timing perceived to be based on service needs. Lack of continuity between inpatient and community services Lack of specialist knowledge in community-based services	Outcomes not discussed
Cotner et al., (2018) Individual Placement and Support for employment	Many participants reported that the job seeking activities yielded positive outcomes (e.g., increased confidence and purpose) even if employment not yet obtained. The authors hypothesized that positive outcomes may be related to intervention activities such as goal setting, community access, increased social networks, individual support and encouragement	Not reported	Contributing to society—giving back, sense of pride Financial independence Improved mood, confidence, self-esteem, purpose, hope New goals set once employment obtained, e.g.,, promotion, increased hours.
Ekelman et al., (2017) Community fitness centre	Supportive community of peers—socialization, support, acceptance, empathy, understanding, encouragement, motivation, positive examples Comfortable and non-judgemental environment for sharing personal and sensitive information, asking questions, advice Trainers: close relationship, positive attitude, provision of resources and advice, feeling like ‘more than just a client’. Compare this relationship favourably with rehabilitation experience. Opportunity for (even small) ongoing improvement	Limited opportunities for interventions and improvement after inpatient rehabilitation: the fitness centre was a rare opportunity, and the only one of its kind in the state	Managing, reducing and preventing body and health problems Improved mood and hope Sense of control, moving forward, routine, accomplishment Social well-being, sense of belonging and acceptance
Folan et al., (2015) Assistive technology (AT) for computer access	Exposure to technology they would not have otherwise encountered Recommend early introduction to AT, to show its potential and integrate into rehabilitation Opportunities to practise skills and gain familiarity	Previous inexperience and negative attitudes towards technology- these perceptions gradually changed. Initially slow and frustrating learning process	Independence in valued tasks and roles, leading to a sense of control, meaning Coping with injury, adjustment Sense of ‘normality’ and self-worth Social interactions Enjoyment and fulfilment from learning something new
Hall et al., (2021) Rehabilitat-ion	Support of family/friends to navigate and access the rehabilitation system, as supports during rehabilitation, and for community participation and reintegration Own positive mindset, hope, self-advocacy, perspective Encouragement and support of rehabilitation providers	Inadequate preparation for discharge Inadequate skills training for community participation, no practical training Lack of indvidualisation Low expectations of health professionals, lack of understanding Too short, lack of follow-up, difficulty accessing programs or funding, lack of specialist services in the community Ongoing environmental barriers limiting community access and participation	This study mostly reported experiences (mostly negative) of rehabilitation: specific outcomes not reported, but some participants described living a good life.
Hitzig et al., (2013) FES and treadmill walking/ exercise training	Valued aspects listed without detail: staff, socializing, program helps the SCI community, organization of the program. Peer support, for education and motivation	Program interfered with other activities, e.g.,, work Travel time inconvenient Program too short	“Gave me back my life” (p. 251) Improved community mobility, social participation Greater independence in daily activities Confidence Improved mood, less fear, e.g.,, of falling
Houlihan et al., (2003) Internet access	Using the internet was an entertaining and interesting pastime, especially compared to previous passive activities, e.g.,, watching TV Able to research information, e.g., about condition, job seeking, transport Internet enabled connections with others, opportunities for meeting new people, staying in touch with existing networks, sharing with others, learning	‘Addictive’ nature of internet	Improved mood Increased meaningful activity options, learning new skills Social connection, support, sharing
Hutchinson et al., (2003) Adapted recreational leisure activities	Enjoyable activities were valued in a hospital setting: diversion, sense of continuity early after injury, sense of personhood (vs being a ’patient’), and increased motivation for rehabilitation program Opportunities to leave the hospital/room, e.g.,, attending a music event, going to a different area to smoke and socialize Meaningful, enjoyable and expressive activities valued, particularly when they restored a sense of self, connection to past identity/values, and connection to others Both passive and active leisure activities important Activities based on a common interest were valued, and took focus away from disability. Shared activities with disabled peers also fostered a sense of belonging for some Importance of engaging in activities that foster a sense of competence, particularly in the absence of roles/identity usually valued by society (e.g., employment)	Not feeling competent was a barrier to leisure participation: effort and embarrassment Social encounters were a negative experience for some	Data analysis focused on coping efforts: Buffer from stressors: escape, relaxation, distraction/ diversion, sense of connection to the past (identity and activities), escape (physical and symbolic), adjustment Motivation to sustain ongoing coping through leisure activities: hope and optimism, structure, purpose, belonging, connection, acceptance, sense of competence and independence, positive identity self-continuity, maintenance of health. Greater community and social participation
Labbé et al., (2019) Community-based adapted recreational leisure activities (RLA)	Feeling adequately challenged was important: a secure environment to push limits and develop new skills Activities that were enjoyable, challenging, meaningful and creative were particularly valued Opportunities to socialize with peers, staff/volunteers, and family/friends during the activities Information provision from program and peers Contact with nature during outdoor activities Importance of planning and customization of activities for accessibility and safety Low cost, variety of programs, links between the program and other community organizations, availability of specialized equipment Expertise and personality of staff members and volunteers	Logistical issues, e.g.,, booking process Transport challenges Limited programs in local area Worried/closed minded family and friends	Recovery, adjustment Sense of continuity Freedom and escape Autonomy: control, independence, making choices Improved mood, relaxation and flow, and physical health benefits Belonging and acceptance, reduced social isolation Reduced stereotypes, positive image Further engagement in other leisure activities and volunteering/employment
Lai et al., (2016) Teleexercise program	Overcoming barriers to exercising at local fitness centres (inaccessible facility or equipment, lack of staff expertise, high costs, limited transport) Convenience, less time taken, flexibility in timing Coaches provided motivation, expertise, monitoring, feedback, and accountability. Technology mostly simple and intuitive	Few opportunities for exercise outside of program Tablet screen used for too small, internet instability (in rural areas)	Increased strength and endurance, less fatigue Increased ability to perform meaningful activities, particularly physical activities
Lape et al., (2018) Adapted sports	More engaging than exercising at a gym Staff expertise, planning/problem-solving and equipment facilitated safe participation and manage risks Taking risks and facing challenges was a source of enjoyment and pride Social relationships with peers and staff: motivation, role models, inspiration, information sharing, opportunity to contribute to others Contact with nature, being outdoors Raised awareness of possibilities by exposure to peers Planning ahead to manage finances, time and energy for participation	Risks of injury, overexertion and exposure (winter sports) Transport consumed energy, time and financial resources Preconceived ideas and low expectations initially limited participation Lack of general awareness of possibilities Lack of awareness about program amongst health professionals Limited program resources Participation limited by personal finances and time	Benefits to physical function, including strength, balance, weight maintenance Improved mood Improved function for daily activities Positive identity, transcending disability, self-continuity Confidence, self-efficacy Further engagement in other sports Expanded world, getting out, making the most of every day
Luchauer & Shurtleff, (2015) Regular physical activity—adapted recreation	Improvements in body functions were a motivator to continue participating Interactions with peers: learning, skill sharing, connection, understanding, socializing Services provide a rare opportunity to engage in accessible physical activity (particularly with access to specialized equipment) The opportunity to work towards something and set goals	Need some level of acceptance of injury in order to participate in adapted sports Support from family/friends required to participate (motivation, transport, and logistics) Funding required for participation Active recreation perceived to be inadequately addressed in rehabilitation: lack of priority, low expectations	Improved strength, fitness and energy Increased ability to participate in daily activities, do enjoyable tasks, decreased burden on families Activities less straining and less risk of injury Improved sense of self, acceptance Social connection
Lucke, (1997) Nursing care during inpatient rehabilitation	Individualized Caring relationship: listening, encouragement, reassurance, humour, mutual respect, interaction/interest on a personal level Risk-taking and ‘breaking the rules’ to meet individual needs (this required knowledge and experience) Being respected as a partner in rehabilitation process, trusted to make decisions and take risks Training and opportunities to practise skills, graded independence Thoughtful decisions about when to try a new task, reduce assistance, try a difficult task again, cease an activity, and provide rest breaks Acting as a consultant as the person gained autonomy, e.g., freedom to experiment with new techniques, advising about risks, creative problem-solving	Inexperienced or casual staff less willing to be flexible, take risks Providing individualized care sometimes required ‘breaking rules’ or going against procedures Defensiveness from some staff about people with SCI trying their own methods/solutions Existing caring relationship was not always considered when staff were assigned to patients Developing a caring relationship takes time, which is usually limited	Reintegration of self Improved mood and hope Greater independence in activities
Maddick, (2011) Music therapy program	Music was an accessible, enjoyable, relaxing activity, and a welcome distraction. Participants looked forward to sessions. Music facilitated expression of feelings, an emotional outlet for negative thoughts/feelings; this was particularly beneficial for adjustment and relationships Safe, non-threatening environment Group setting with people they could relate to, shared experience and support, expanded musical experiences. Privacy in individual sessions also valued Opportunity for creative expression, realization of talents	Opportunities for group support were not otherwise provided in rehabilitation Limitations of program not reported	Confidence, self-esteem Greater ability to perform activities, new accessible activities related to music, sense of pride and achievement Improved mood Adjustment, hope Improved relationships, benefits to families Pain management Physical gains, e.g.,, finger function, voice/breathing Greater participation in other rehabilitation therapies
Mattar et al., (2015) Existing use of IT and specialist access equipment	Equipment and modifications enabled access to IT, e.g.,, mounts, adapted mouse devices, voice recognition software. IT became invaluable, kept close at hand Used for managing schedules, researching information, work tasks, planning events and activities, researching and managing health and physical activity. Facilitated connection with existing and new networks, providing socialization, information, support, motivation AT departments in rehabilitation exposed people to devices and access options	Not all IT devices were accessible, use sometimes caused pain/fatigue Previous negative experiences with old technology, frustrations, e.g.,, voice recognition Concerns about future technology: design ‘enhancements’ can decrease accessibility Lack of IT experience, training/learning process frustrating Cost of IT a barrier Information found on-line not always reliable, sometimes research caused anxiety Concerns about on-line security Social media exposure can lead to feelings of exclusion Concerns about spending too much time using IT, ‘dependence’	Control and independence in activities and routines, community access Ability to perform tasks from home and more flexibly (e.g., work) Social connection
Nygren-Bonnier et al., (2018) Glosso-pharyngeal breathing training	Participants valued learning a new technique and having increased awareness of, and control over, their breathing Access to ongoing training and expertise helped with the learning process	Negative reactions of others when using a non-conventional breathing technique Learning the technique was challenging and stressful, with benefits not immediately obvious: cost vs benefit was questioned (at least initially) Side effects included dizziness, fainting, sense of bloating, tingling	Improved lung function (easier, deeper ventilation, more efficient expiration), cough efficiency, voice and sleep Benefits to balance, fitness, endurance Physical benefits resulted in improvements to mood, sense of agency, hope, greater endurance for activities
O’Dell et al., (2019) Peer support program	Shared experience: role model, inspiration, demonstrating possibility of a good life post-SCI Legitimacy for challenging conversations Information provided in a way they could identify with Health professionals felt peer support supplemented their own interventions and also valued training they received from the peer support workers Family members valued advice too and were sometimes willing/ready to talk before the person with SCI	Occasional personality clashes Some uncomfortable asking intimate questions of a peer of opposite gender Not always ready to talk or knowing what to ask early on Post-discharge support valued but lacking	Reduced isolation of the person with SCI and their family/friends Greater awareness of the situation and possibilities Increased knowledge
Ramakrishnan et al., (2016) Early intervention vocational program	Most valued early timing of intervention: awareness of options, direction, more likely to be interested Advocacy to employers Integration of employment goals into general rehabilitation Care, compassion, innovation and efficiency of vocational consultants Provision of information and resources	Some felt ICU/acute setting was too early: not a priority, ‘invasive’, dealing with health and lots of other information, too much uncertainty Wanted better communication about the role of vocational consultants Need for services later on if not ready to pursue work early after injury	Hope early after injury, adjustment Early positive expectations, confidence and motivation Improved mood, distraction from problems Feeling empowered Inspiration and direction to work on other goals, e.g.,, driving Self-esteem, continuation of a vocational identity
Semerjian et al., (2005) Adapted exercise program	Particularly valued the body-weight support system, which facilitated standing and walking Some found the aerobic exercise trainer (active passive trainer) enjoyable “I get in the flow, you know, the zone … seems like you can go on forever” p102.	Inability to get set up on/use some equipment independently due to impaired grasp Self-consciousness in harness, especially as it emphasized ‘gut’ (lack of abdominal tone) Wanted opportunity to continue after sessions/program	Increased strength, endurance, energy, better gait and trunk control, increased/less spasticity Increased satisfaction with appearance Increased capacity to perform activities and maintain activities with less fatigue, go out more Improved mood Emotional benefits of (supported) standing and walking: fun, sense of self and normality, sense of perspective and height when standing near others, dignity Sense of hope from warding off problems, being in a position to take advantage of future treatment advancements
Singh, Shah et al., (2018) Mobility training program	Valued opportunity for higher intensity training, compared to existing outpatient rehabilitation: desire to maximize potential/gains early on. Program customized to individual needs Educational component—increased knowledge Valued having a structured program/routine soon after discharge Rapport and collaborative relationship with the clinicians: looked forward to attending, sense of friendship, and belonging Development of measurable goals to monitor progress Supportive equipment setup, e.g.,, treadmill harness enabled a safe environment to learn skills and take risks Transfer of skills from treadmill to real outdoor environments	Limited opportunities for ongoing intervention outside of the research Balancing time of program with other valued/important tasks Long travel distances to program, reliance on carers for transport Extra support needed for participants with incontinence Wanted greater transfer of skills to real-world environments Wanted more flexibility and challenge once skills were mastered Need for falls education Some felt the intense program was exhausting	Hope Increased strength and endurance, resulting in improved mobility and independence in activities Sense of empowerment and control from increased knowledge Improved mood Greater confidence, self-efficacy
Singh, Sam et al., (2018) Mobility training program—long term follow up from above study	Structure and routine eased transition home Emotional support from clinicians Resources provided about longer term opportunities and home exercise programs	Sense of disappointment when program ended, desire to engage in ongoing opportunities (limited) and develop new routines Long-term desire to continue making gains and preventing decline—mostly this was through community gym or home-based exercises	Increased strength, resulting in greater independence Able to engage in activities without overexertion Confidence Better sleep Worsened mood and hope when program ended, but this eventually improved for most participants
Swaffield et al., (2021) Activity based therapy	Sense of normalcy: setting was ‘like a gym’ Sense of community and acceptance, opportunity to interact with others with SCI Clinicians focused on possibilities rather than limitations; were open to experimenting and new ideas, but were also realistic about potential Appreciated the high intensity, individualized programs Some participants found ways to engage in similar activities outside of the clinic, e.g.,, at local gym, although ability to progress and social interaction were lacking	Participants reported a lack of priority for this type of therapy in rehabilitation, which focused on compensatory interventions and exercises above the level of injury Mental effort required was tiring and frustrating (although seen as necessary) Time commitment was challenging, but seen as a priority. Few services available. Cost of therapy, insurance funding not always available Varying levels of skill amongst the clinicians Some injuries reported Lack of awareness/referral from rehabilitation professionals; negative outlook/low expectations	Improved independence in activities, ability to live alone, community and social participation Improved neurological function, e.g.,, strength, sensation Improved health, e.g.,, cardiovascular fitness, and decreased secondary health conditions Active lifestyle Improved mood, reduced stress and depression Confidence, positive outlook Hope Improvements a part of a gradual and long-term process; rehabilitation seen as lifelong
Tamplin et al., (2014) Group singing/music appreciation	An enjoyable, accessible and meaningful activity Socialization, sharing and peer support in groups; sense of safety, support, belonging and inclusiveness Sessions provided a reason to access community, and get out of bed earlier.	Greater insight into voice issues were initially challenging Nervous singing in front of others Meeting in a group with others with a disability was a confronting reminder of disability for some	Improved mood Confidence and hope Greater appreciation of music and its effect on mood, reconnection to past interests Improved energy, relaxation, sleep, pain Experience of flow Improved vocal quality and breathing
Taylor & McGruder, (1996) Sea kayaking expedition with recreational therapist	The experience of being in nature A fun, relaxing, enjoyable activity Social interaction with peers with SCI, with a focus on a shared activity rather than disability. Support and encouragement, new friendships Novelty of the activity was positive and helped with ‘moving on’ Overcoming initial low expectations, meeting challenges, redefining limits and self-perceptions An opportunity to apply skills learnt in rehabilitation setting	Rehabilitation focused on regaining old activities not on engaging in new ones, which could be confronting/frustrating Lack of awareness amongst rehabilitation staff about non-traditional activities Desire for more opportunities to engage in similar activities	Increased social interaction and new friendships Coping, adjustment, stress management Meaning and routine Self-esteem, confidence Improved strength and endurance Improved mood
Veith et al., (2006) Peer mentoring program	Information, role modelling and inspiration to counter initial fears, uncertainty and low expectations Detailed practical information that was not provided by professionals Downward comparisons helped foster a sense of appreciation Most appreciated having a mentor who was slightly older, of the same gender and with an equivalent injury level Aspects of the relationship: informal, casual relationship with a social and friendly mentor, humour and positive outlook Shared experience meant the mentor was a trusted and credible source of information. Sense of understanding, equality, acceptance; normalizing their experience and reactions	Mentoring appeared less important for people with an internal locus of control, and/or strong family support Logistical issues meant people had fewer meetings than desired Significant age differences affected mentoring relationship for some	Hope, positive expectations Reduced distress and fear
Verdonck et al., (2011)	Perceived outcomes were discussed, not experiences of the intervention itself	Not reported in this study. Half of the participants did not yet have access to an ECS “as a result of circumstance (not choice)” p272.	Time alone: privacy, space Changed relationship dynamics: less dependence, able to contribute, less perceived annoyance Reduced care hours for some participants Less worry for carer and people with SCI Feeling more confident to be at home/go out alone
Verdonck et al., (2014) Loan of environmental control system (ECS)	The intervention provided new opportunities to participants with long-term SCI, who had accepted their need for assistance and had not initiated seeking alternatives With practise, effort and experimentation, participants learnt to use the system Using ECS enjoyable and addictive Surprise about their ability to use the system, its potential, and the enjoyment of engaging in new tasks	Adjusting to the new system required effort, required new routines and habits Frustrations with the system included complexity/inefficiency of switch scanning (vs asking a carer for help) and technical issues ECS not routinely considered in rehabilitation, funding limitations	Positive emotions Engagement in new roles Ability to engage with others in a fun and spontaneous way Increased control and choice Independence and privacy
Verdonck et al., (2018) Loan of ECS	Even small gains in independence had a big impact on life and emotions, in the context of being able to do very little otherwise Simple everyday tasks were valued, e.g.,, changing TV channel, turning on a light, and answering phone calls Independence in these tasks was pleasurable and had symbolic meaning Increased independence in some tasks and ability to call for assistance, meaning that carers could be more distant Able to accomplish tasks, which would have otherwise been neglected in an attempt to reduce perceived carer burden	Carers still needed to be readily available Participants accustomed to assistance of other people, and did not feel confident or safe to reduce hours of care	Reclaiming previous abilities Improved ability to make spontaneous choices and sense of freedom Reduced reliance on assistance resulting in improved relationship dynamics: reduced frustration (and perceived reduction in frustration for carers), reduced sense of burden and obligation Increased privacy, able to be alone, enjoy own company and ‘peace and quiet’ Increased sense of safety and security Improved mood, positive emotions Enhanced sense of self
Wangdell et al., (2013) Reconstructive hand surgery	Positive outcomes were discussed, not experiences of the intervention itself	Researchers asked about negative experiences but few were reported Some reported thumb stiffness affecting grasp soon after surgery	Improved self-efficacy in hand control, leading to enhanced independence New activities made possible, mobility and exercise activities easier (e.g., grasping gym equipment), tasks ‘smoother’ and quicker, less reliance on compensatory methods, less impacted by environmental barriers Improved participation Reduced reliance on assistance: able to be alone longer, reduced care hours, able to perform a task rather than waiting for help Privacy, enjoying own company, able to carry out private tasks without assistance Confidence and control, self-esteem Regained identity as active, independent, social and equal Reclaiming part of the body they missed Improved relationships from increased ability to engage in activities, less need for assistance, ability to contribute, reduced sense of burden, and a shared experience of hope and improvement (vs shared sense of loss after injury) Initial successes led to seeking out further challenges and new occupations
Ward et al., (2007) Occupation-based OT interventions	Identifying and practising valued occupations, which were related to previous interests, self-identity and roles Experiencing performing valued occupations helped participants (and family) realize they could still engage in these occupations, even if in a different way Involvement of family and friends in therapy sessions were valued by both parties, and family/friends were a source of motivation Therapy sessions (and related occupations) were an opportunity for self-expression, enjoyment, interest, engagement and escape. Sessions felt like ‘real life’ rather than therapy. Collaborative problem-solving, teaching problem-solving skills, exposure to a range of techniques and solutions Positive ‘can do’ attitude of staff, which countered initially low expectations Exposure to varying, real environments provided a helpful experience and mindset for ongoing participation in the community Provision of information about community-based resources for longer-term/specialized support, e.g., adapted skiing organization Providing practical support and follow-up for unfamiliar tasks, e.g.,, applying for social security Facilitation of community occupations through home and vehicle modifications and skills training: these occupations were particularly valued, providing a sense of escape, socialization and identity as a community member/contributor	Not reported	Ability to engage in valued occupations, maintain roles and contribute to the community Retained/restored sense of identity Positive mindset and expectations Self-efficacy in solving own problems Increased community and social participation
Wellard & Rushton, (2002) Inpatient pressure ulcer management	Access to windows and outdoor areas helped patients feel more connected to the outside world Social activities facilitated by staff enabled time with family/friends outside of the ward environment	Space less flexible and personalized than at home, limiting independence Spatial practises were perceived to prioritize efficient use of resources over patient interests/experience Lying in bed increased a sense of dependence and helplessness Distance from home to hospital, limited, inflexible visiting hours, and lack of space for visitors impacted families: drain on energy and finances, some members unable to visit, family members left out of information and decisions Unpleasant physical environment affected mood Lack of privacy (shared rooms) for personal conversations, personal care routines Feeling disconnected, confined, punished Lack of opportunity for social connected (limited access to telephone) and intimacy Fear of these negative experiences often delayed the decision to seek treatment for pressure injury	Reduced independence, compared to home environment Reduced sense of personhood and redefined identity as ‘sick, disabled other’ Negative impacts on mood: sense of confinement and dependence, depression Reduced social connection, impact on family relationships
Williams et al., (2014) Leisure time physical activity	Key motivators for participation included a desire for greater independence, less need for assistance, being in a position to take advantage of future treatments/cure, fulfiling valued roles and contributing to society. Participating with peers with SCI raised awareness and expectations and was an opportunity for learning and socialization. Information about accessible opportunities mostly provided by peers, and occasionally health professionals (who learnt about these opportunities from their patients) Experience of supported walking/standing was enjoyable, provided dignity, and a momentary return to ‘normality’	Low levels of subjective well-being and social participation (e.g., reduced confidence, fear of exclusion) were barriers to physical activity Environmental barriers to participation included finances, high cost of participation/equipment, lack of accessible facilities, cold weather for outdoor activities, transport, dependence on assistance of others to exercise (and lack of this assistance) Body problems (e.g., fatigue and fear of injury) were barriers to participation Lack of information about accessible opportunities (including amongst health professionals) Limited time, energy and motivation outweighed the limited benefits for some people Perceived negatives of disabled sports: some did not enjoy modified versions of their previous sports, inability for able-bodied friends to participate, some women felt in the minority, and some associated it with unhealthy masculine behaviour Authors caution against overemphasis on individual responsibility for physical activity whilst ignoring environmental barriers, overemphasis on sport at the expense of other expressions of self and masculinity, and potential negative outcomes if hope for cure/recovery is the sole motivator for participation.	Improved subjective well-being (e.g., mood), psychological well-being (outlook, purpose) and social well-being. Improved body functions (e.g., pain, strength, and fitness) Improved body-self relationship, identity, sense of self Positive cycle: these positive outcomes acted as motivators for ongoing participation
Zinman et al., (2014) Self-management education program	Greater understanding of the role of self, need for assertiveness and advocacy Adjustment facilitated through greater insight into own limitations, need to accept limitations and communicate these to others, and understanding of potential Knowledge gained through education and group interaction, skill acquisition, self-management strategies, access to resources Implementing and practising skills during supported community outing Experience of goal-setting and pursuit led to new goals being set Group dynamics and supportive environment with peers: sharing experiences and knowledge, comparisons, able to relate to each other.	Wanted longer and more sessions and a follow-up service	Development of post SCI identity improved self-esteem, confidence Adjustment, positive outlook, hope, gratitude Increased community participation

Valued aspects included positive expectations of service providers, which raised the expectations of people with SCI about what was possible: “*She just kind of conveyed this feeling to me … that I was going to be able to do just whatever*” (Ward et al., 2007) p.153. The personal characteristics of service providers were also important in facilitating a positive, supportive environment, and valued staff attributes included respect, recognizing dignity and equality, warmth and friendship. “*she was so reassuring and she was so caring and so pleasant, and there to tell me, okay, I—I’m not alone*” (Ramakrishnan et al., 2016) p.188. Peers with SCI were another source of positive expectations and hope: *“ … opens your mind up to all the things you can do and the way that you can get around it*” (Beauchamp et al., 2016) p.1980. Peer support (whether formal or incidental) was also valued for the connection, belonging and understanding brought about by interaction with people in a similar situation: “*It’s a great way to have a bit of camaraderie and a feeling of group, a sense of being in a group or a community*” (Tamplin et al., 2014) p.241. People with SCI valued long-term opportunities for continued gains and improvement (in a range of areas), even if these gains were seemingly small. They also wanted opportunities to challenge themselves and take risks, although the right level of challenge was important: experiencing too many difficulties was confronting and discouraging. Services that facilitated participation and autonomy in meaningful occupations were highly valued, including in both pre-injury and new occupations: *“ … makes you feel good because it does feel the same as before [the accident]*” (Tamplin et al., 2014) p.240. Learning new skills helped improve autonomy, and people with SCI wanted practical and applicable information that enhanced this learning. They also valued having their own problem-solving skills recognized and enhanced: an important way to gain autonomy in the long term. Their own efforts, character and determination were also crucial: *“ … what helped me the most was my own will to be independent*” (Bernet et al., 2019) p.6. Connections with others were important, and services were valued when they facilitated interaction with peers and provided opportunities to engage with significant others: “ … gives me an opportunity to do something together that we both like” (Hutchinson et al., 2003) p.152. People with SCI noted that the contribution of supportive family and friends in facilitating participation in valued activities and the support of others (e.g., transport to clinics) was often crucial for being able to participate in services.

There appeared to be an important balance between setting positive expectations and facilitating achievement early after injury, whilst not overwhelming the person during the acute stages post-SCI. People with SCI expressed varying perspectives about preferred timing of services, and flexibility in delivery appears important (although not always provided). Discharge home was a challenging milestone, and people with SCI valued services in preparation for, and soon after, discharge. Community-based services provided soon after discharge provided structure, routine, and an opportunity to maintain or continue gains made in an inpatient setting “*I am feeling like I am accomplishing something throughout the day*” (Ekelman et al., 2017) p.34. There did not appear to be a time that was too late to provide services, and gains were valued even many years post-SCI. However, opportunities to engage in services at this stage were rare. The physical environment of services was important to people with SCI. Opportunities to be in (or at least see) nature and the outdoors were highly valued: “*you get excited about nature, clouds and the currents …* ” (Taylor & McGruder, 1996) p.42. An unpleasant, inflexible, impersonal hospital environment had a negative impact on well-being in one study.

Overall, people with SCI reported few limitations of the services themselves, but a common theme was a lack of opportunity to participate in well-being-related services. Some of the services were provided only during the study period, and people with SCI often reported that they wished the services were longer or more available outside of a research context. Many people with SCI reported a lack of opportunity for accessible activities that promote well-being, particularly in a community setting or many years after injury. When suitable services were available, travel costs and logistics to access the services were often challenging or prohibitive. People with SCI were often made aware of community-based services by peers or through their own research, and there was a perceived lack of awareness of such opportunities amongst health professionals: “*But it seems there really isn’t an awareness when you have to explain what you want and what you’re going to do and they just look at you like ‘really, you’re going to do what?*’” (Taylor & McGruder, 1996) p43. Although a number of studies described services provided during inpatient rehabilitation, some people with SCI reported a lack of priority for such activities during their own early rehabilitation journey. Low expectations of health professionals and inflexible service delivery were limitations of inpatient rehabilitation reported by some study participants.

People with SCI reported a range of service outcomes, which related to both psychological and subjective well-being. Participants reported improved confidence and self-esteem, coping strategies, motivation, sense of identity, and normality. Improved mood, positive emotions, and sense of gratitude were also reported. “*I feel much more alive. Enlivened and engaged with what I’m doing”*(Tamplin et al., 2014) p.241. “*It just helped to show us that there’s still a hell of a lot that we can be thankful for*” (Zinman et al., 2014) p.9. A commonly reported outcome was increased hope, which participants felt was especially important to their well-being: “*I think that was the hope that actually even helped me to get better*” (Ramakrishnan et al., 2016) p.188. People with SCI reported improved independence and autonomy in performing occupations, which brought about greater control choice, privacy, and flexibility in their day-to-day lives. Greater autonomy enabled people with SCI to contribute to others and perform valued roles and reduced their sense of burden on others, frustration and ‘hassle’: “*the more independent I will be and the more I can do for others … that’s gonna make me feel so much better with myself*” (Semerjian et al., 2005) p.101. The subjective experience of performing occupations (of any kind) involved a sense of fun, enjoyment, flow, engagement, meaning, purpose, freedom, escape, diversion and relaxation: “*You create some endorphins, and you’ve got your circulation working better … it’s really had an effect on my whole outlook*.” (Lape et al., 2018) p.509. Increased autonomy in one occupation often had a flow-on effect, with participants often setting new goals, trying new activities, and having greater motivation to participate in other rehabilitation activities: “*It gave me the motivation to stay with the rest of the therapy*” (Hutchinson et al., 2003) p.152. People with SCI also reported improvements in relationships and a sense of belonging, as services enabled them to participate more in the community, provided new social contacts, and facilitated a sense of belonging. Perceived outcomes of a sub-set of the included studies are explored in more detail in a separate publication (Simpson et al., 2020).

## Discussion

A contribution of this scoping review is the synthesis of qualitative research from a variety of disciplines, which readers may not have encountered otherwise. People with SCI reported well-being outcomes from a range of service types, reflecting the multidimensional nature of well-being and the fact that its determinants are relevant to a range of disciplines. Well-being may be addressed from different perspectives, and it is important for service providers to recognize their own potential to influence well-being, as well as the contribution of other team members. Improving well-being can and should be a common aim, which may require rehabilitation professionals to broaden their focus beyond the discipline ‘silos’ that may still exist in rehabilitation.

We argued earlier that a more explicit focus on well-being may maximize the impact of services on the lives of people with SCI, echoing calls from prominent rehabilitation researchers (Hammell, 2006, 2017; Pizzi & Richards, 2017). One of our aims was to explore how well-being was conceptualized in the included studies, and whether the services were intentionally designed to address well-being. Most authors did not define well-being (or related term). In several studies, improved well-being appeared to be a finding rather than an aim of the service. Four studies included a broad definition of well-being that appeared to be a helpful framework for service design and evaluation. However, we were not able to determine whether or how well-being frameworks were used to design the services because information about service design was rarely provided, possibly due to word restrictions. The question remains, does intentionally designing services to address well-being elements produce greater impacts on well-being? Or is well-being so broad that services can impact it without intentionally aiming to do so? Further research is needed to shed light on these questions, which our review seems to have highlighted. Such research may include evaluation of programs that deliberately aim to address well-being by targeting its determinants. A review of the quantitative literature would also be helpful, particularly in shedding light on the question of whether a more explicit focus on well-being produces greater well-being outcomes.

This scoping review has synthesized important insights from people with SCI about valued aspects of services. These insights may inform service design and evaluation. A key finding was the importance of the characteristics and approach of service providers, including respect for the autonomy and dignity of people with SCI. Services that facilitated autonomy and control were valued, and these influenced well-being in a number of ways. People valued having their own skills and strengths recognized and encouraged, such as problem-solving skills. These skills are important for self-care and self-management after SCI, and an important way people with SCI can manage the impact of their condition on well-being (Conti, Clari et al., 2020). Psychological strengths and resources are an important well-being determinant (Clifton et al., 2018; Peterson & Seligman, 2004; Simpson et al., 2020). Interestingly, we did not find any qualitative studies from the psychology literature. Such studies may provide valuable insights about how psychological strengths can be identified, recognized and nurtured to promote well-being. Recognition of the importance of the skills and behaviours of people with SCI is congruent with the literature about self-care and self-management

Another key theme was the importance of positive expectations. Service providers who promoted high expectations and facilitated hope influenced well-being by countering the low expectations that people may have initially held about the possibility of a good life. Hope was also important to people with SCI, and increased hope was a valued outcome of some services. Hope and positive expectations did not appear to relate to a potential cure for SCI (although some participants discussed physical activity as a way of taking advantage of a future cure). People found hope in a good life in the absence of a cure, and despite the presence of significant impairment. This finding is consistent with the social model of disability that environmental factors are a vital influence on well-being (Barnes, 2019; Hammell, 2007; Oliver & Sapey, 1999). However, well-being can also be influenced by body functions, and people with SCI reported important well-being outcomes when body problems such as pain and fatigue were addressed. They also reported many persistent environmental barriers that limited their ability to participate in well-being promoting activities and services. Spinal cord injury challenges simplistic distinctions between the medical and social models of disability, revealing how well-being is embodied. It is always a product of the complex interplay between bodily function and the social environments (Mackenzie & Scully, 2007; Siebers, 2008).

The importance of interaction with peers with SCI was another key theme, and this related to the theme of positive expectations. Being exposed to the life of a person with a similar injury raised expectations about what was possible. Peer interaction was also an important source of social contact, belonging and understanding. Several of the studies involved formal peer support services, and these services appear to strongly influence well-being, particularly soon after SCI when a person may be unsure about what life may hold. However, peer contact was also provided incidentally by many of the services and facilitating informal peer interaction may be an important way service providers can influence well-being. Although beyond the scope of this paper, this peer emphasis suggests spinal cord injury services should give more thought to the importance of coproduction in the design and delivery of programs (Alakeson et al., 2013; Ryan, 2012).

There appear to be limited opportunities for people with SCI to participate in services to improve well-being, particularly in the community or many years post-SCI. Rehabilitation services often end within several years of injury, with longer term follow-up often focusing on managing problems like pressure injury and replacing old equipment rather than improving well-being. Interestingly, the majority of studies in this review involved community-based services, with several provided to people more than 20 years post-SCI. These studies contributed valuable insights from people many years post-injury. However, it appeared that many of these services were provided for the purposes of research, rather than being available generally or in the long term. Several participants reported a lack of services available to them or that they wanted services to be available beyond the study period. Ideally, services for people with SCI would promote sustainable change and autonomy, so that they are not required long term. Presumably, most people with SCI would prefer to become independent of specialized services if possible, although ongoing physical problems such as pain, and the effects of ageing with SCI, may necessitate some long-term specialized input. But people with SCI did value services provided to them many years post-injury and reported well-being outcomes from such services. Some of these services involved learning a new skill, e.g., a breathing technique, and trialling new assistive technology. If similar services are not provided outside of a research context, people with SCI might be missing out on exposure to new techniques and technology, especially when they are no longer involved with a formal rehabilitation service. Several of the community-based services were ongoing, involving adapted sport/exercise and recreation. It was clear that many people with SCI required long-term and specialized services to engage in these activities, presumably due to their needs (e.g., access requirements) not being met by mainstream services. However, gaps in such services were also reported, with participants reporting lack of services in their area or limited program resources.

These gaps may reflect the significant funding and insurance limitations that constrain the provision of services to people with SCI. SCI service providers may need to be creative and resourceful in order to offer well-being services outside of a traditional rehabilitation context. Research on the feasibility and outcomes of such services may also expand our understanding of how well-being can be improved across the lifespan of people with SCI.

## Limitations

The studies in this review predominantly included the voices of people with SCI from USA and Canada, and findings may most strongly reflect the intervention context in North America. We did not include studies published in languages other than English, so may be missing the perspective of people from non-English speaking countries, whose experiences of SCI, services and well-being may differ from those in this review. Some studies included people with a range of conditions, and we were not able to distinguish which findings specifically related to participants with SCI. However, services are not always limited to people with SCI, and further research on whether and how services can impact well-being of people with a range of conditions would be worthwhile. Our key finding about peer support would also be interesting to explore in the context of these broader services, where the concept of ‘peer’ may extend to people with different diagnoses and conditions.

Methodological issues in the included studies may have impacted our findings. Some studies did not report many negative experiences or outcomes, and the absence of interview guides meant that we were not always able to determine whether this information was sought. The background and position of researchers is a potential source of bias, and for many studies we were unable to determine if and how biases were identified and managed. There did not appear to be much (if any) contribution of people with lived experience of disability as co-researchers (Mellifont et al., 2019). The absence of this perspective may have been a source of bias in design and analysis.

Our own backgrounds and perspectives have influenced design and analysis. SC is an academic with spinal cord injury, who has researched factors that affect the flourishing of people with disabilities. MV and BS have an occupational therapy background, which contributed expertise about (and a bias towards) occupation-related findings. MV’s research is characterized by large-scale collaborations that cross disciplines and sectors and that privilege the voices of those typically marginalized within research. The distinguishing feature of this research is our intentional application of inclusive models and participatory methodologies to bring people who do not normally work together to solve complex problems through cross-sector collaboration and co-production.

## Conclusion

This scoping review has identified qualitative studies from a broad range of disciplines, who seek to address well-being from a variety of perspectives. A strong conceptual framework of well-being is mostly lacking in this body of literature, despite calls for a more explicit focus on well-being in rehabilitation services. Despite this, people with SCI reported a range of well-being outcomes. Valued aspects of services included a positive and empowering approach of service providers, the opportunity to participate in and gain autonomy in valued occupations, and peer support and interaction. However, many people with SCI reported a lack of such services available to them, particularly after inpatient rehabilitation.

## Supplementary Material

Supplemental Material
