# Supplementary material for: Exploring well-being services from the perspective of people with SCI: A scoping review of qualitative research
Source: Int J Qual Stud Health Well-being. 2021 Oct 25;16(1):1986922. doi: 10.1080/17482631.2021.1986922 (PMC8547844; doi:10.1080/17482631.2021.1986922)
Supplement: Supplemental Material [file ZQHW_A_1986922_SM1209.docx]

Critical Appraisal Skills Programme (CASP) qualitative checklist

| **CASP section** | 1- Aims | 2- Methodology | 3- Design | 4- Recruitment | | 5- Data collection | | | | | | 6- Researcher- participant rel. | 7- Ethical issues | | | 8- Data analysis | | | | 9- Findings | |
| --- | --- | --- | --- | --- | --- | --- | --- | --- | --- | --- | --- | --- | --- | --- | --- | --- | --- | --- | --- | --- | --- |
| **First author** | Clear statement of aims?  **CASP hints** | Qualitative methodology appropriate? | Research design appropriate/justified? | Explained participant selection? | Discussion of recruitment issues? | Setting/methods clear? | Methods justified? | Methods explicit e.g. interview guide? | Discussion of modification of methods? | Form of data clear? | Data saturation discussed? | Examined own influence on recruitment, design? | Adequately informed consent? | Discussion of ethical issues raised by study? | Ethical approval | In-depth description of analysis, reporting? | Clear how themes derived from data? | Sufficient data? | Critical examination of own bias/influence ? | Findings explicit, related to research questions? | Methods to enhance credibility? |
| Beauchamp (2016) | + | + | + | + | ? | + | + | - | ? | + | - | - | + | ? | + | + | + | + | - | + | + |
| Bernet (2019) | + | + | + | + | ? | + | + | - | ? | + | - | - | + | ? | + | + | + | + | - | + | + |
| Block (2010) | + | + | + | + | ? | + | + | + | ? | + | - | - | + | ? | + | + | + | + | - | + | + |
| Brillhart (1997) | + | + | + | - | ? | + | + | - | ? | + | - | - | + | ? | + | + | + | + | - | + | + |
| Chemtob (2018) | + | + | + | + | ? | + | + | + | ? | + | + | - | + | ? | + | + | + | + | + | + | + |
| Conti (2020) | + | + | + | + | ? | + | + | + | ? | + | + | - | + | ? | + | + | + | + | + | + | + |
| Cotner (2018) | + | + | + | + | ? | + | + | - | ? | + | - | - | ? | ? | + | + | + | + | - | + | + |
| Ekelman (2017) | + | + | + | + | ? | + | + | + | ? | + | + | - | + | ? | + | + | + | + | + | + | + |
| Folan (2015) | + | + | + | + | ? | + | + | + | ? | + | - | - | + | ? | + | + | + | + | + | + | + |
| Hall (2021) | + | + | + | + | ? | + | + | + | ? | + | + | - | + | ? | + | + | + | + | - | + | + |
| Hitzig (2013) | + | + | + | + | ? | + | + | - | ? | + | - | - | + | + | + | + | + | + | - | + | + |
| Houlihan (2003) | + | + | + | + | + | + | + | + | ? | + | - | - | + | ? | ? | + | + | + | - | + | - |
| Hutchinson (2003) | + | + | + | + | ? | + | + | + | + | + | - | - | ? | ? | ? | + | + | + | - | + | + |
| Labbé (2018) | + | + | + | + | ? | + | + | + | + | + | - | - | + | ? | + | + | + | + | + | + | + |
| Lai (2016) | + | + | + | + | ? | + | + | + | ? | + | - | - | + | + | + | + | + | + | - | + | + |
| Lape (2018) | + | + | + | + | ? | + | + | + | ? | + | - | - | + | ? | + | + | + | + | - | + | + |
| Luchauer (2015) | + | + | + | + | ? | + | + | + | ? | + | + | - | + | ? | + | + | + | + | - | + | + |
| Lucke (1997) | + | + | + | + | ? | + | + | - | ? | + | - | - | + | ? | ? | + | + | + | - | + | + |
| Maddick (2011) | + | + | + | + | + | + | + | + | ? | + | - | - | ? | ? | N/A | - | + | + | + | + | + |
| Mattar (2015) | + | + | + | + | + | + | + | + | ? | + | + | - | + | ? | + | + | + | + | - | + | + |
| Nygren-Bonnier (2018) | + | + | + | + | ? | + | + | + | ? | + | - | - | + | ? | + | + | + | + | - | + | + |
| O’Dell (2019) | + | + | + | + | ? | + | + | - | ? | + | - | - | + | ? | + | + | + | + | - | + | - |
| Ramakrishnan (2016) | + | + | + | + | - | + | + | + | ? | + | + | + | + | ? | + | + | + | + | - | + | + |
| Semerjian (2005) | + | + | + | + | ? | + | + | + | ? | + | - | - | + | ? | + | - | + | + | - | + | + |
| Singh (2018a) | + | + | + | + | ? | + | + | + | ? | + | - | + | + | ? | + | + | + | + | + | + | + |
| Singh (2018b) | + | + | + | + | ? | + | + | + | ? | + | - | - | + | ? | + | + | + | + | - | + | + |
| Swaffield (2021) | + | + | + | + | ? | + | + | + | ? | + | + | - | + | ? | + | + | + | + | - | + | + |
| Tamplin (2014) | + | + | + | + | ? | + | + | + | ? | + | - | - | + | ? | + | + | + | + | - | + | + |
| Taylor (1996) | + | + | + | + | ? | + | + | + | ? | + | - | - | + | ? | ? | + | + | + | - | + | + |
| Veith (2006) | + | + | + | + | ? | + | + | + | ? | + | + | - | + | ? | + | + | + | + | - | + | + |
| Verdonck (2011) | + | + | + | + | + | + | + | + | ? | + | - | - | + | ? | + | + | + | + | + | + | + |
| Verdonck (2014) | + | + | + | + | ? | + | + | + | ? | + | - | - | + | ? | + | + | + | + | + | + | + |
| Verdonck (2018) | + | + | + | + | ? | + | + | + | ? | + | - | - | + | ? | + | + | + | + | + | + | + |
| Wangdell (2013) | + | + | + | + | ? | + | + | + | + | + | - | - | + | ? | + | + | + | + | + | + | + |
| Ward (2007) | + | + | + | + | ? | + | + | + | ? | + | - | + | + | ? | + | + | + | + | - | + | + |
| Wellard (2002) | + | + | + | + | ? | + | + | + | ? | + | - | - | + | ? | + | + | + | + | - | + | + |
| Zinman (2014) | + | + | + | + | ? | + | + | + | ? | + | + | - | + | ? | + | + | + | + | - | + | + |

+ reported

- not reported

? not reported, but may have been N/A

Note: only primary research analysed
